# Early warning on safety risk of highly aggregated tourist crowds based on VGGT-Count network model

**DOI:** 10.1371/journal.pone.0299950

**Published:** 2024-03-28

**Authors:** Jingjing Liu, Gengan Wu, Yao Liu

**Affiliations:** 1 College of Tourism, Huaqiao University, Quanzhou, Fujian, China; 2 Quanzhou Bolang Technology Group Co., Ltd, Quanzhou, Fujian, China; Zhejiang University of Technology, CHINA

## Abstract

In the era of mass tourism, more and more people are attracted by internet-famous site. With people’s demand for travel surged, tourists are getting together in one scenic spot with doubling numbers, which easily leads to high concentration of tourists with uncontrollable security risks. It needs to be highly valued by the tourism department. Monitoring and issuing warnings for crowd density in scenic areas with Highly Aggregated Tourist Crowds (HATCs) is an urgent challenge that needs to be addressed. In this paper, Highly Aggregated Tourist Crowds is taken as the research objective, and a VGGT-Count network model is proposed to forecast the density of HATCs. The experimental outcomes demonstrated a substantial improvement in counting accuracy for the ShanghaiTech B and UCF-QNRF datasets. Furthermore, the model allows for real-time monitoring of tourist attractions, enabling advanced prediction of high concentrations in scenic areas. This timely information can alert relevant authorities to implement preventive measures such as crowd control and flow regulation, thereby minimizing safety hazards.

## 1 Introduction

With the arrival of the era of mass tourism, people’s demand for tourism has surged, especially in certain holidays and scenic spots, where tourists are highly gathered, which easily leads to uncontrollable security risks. Tourist destinations with high concentration of tourists significantly impact tourists’ travel experiences, making it challenging to ensure their personal safety [1]. The combination of large crowds and their constant movement creates the potential for various incidents like overcrowding and stampedes, leading to extensive damages and casualties [2]. For example, in the stampede on the Bund in Shanghai in 2014, 310000 people gathered in the square for only 10 minutes, resulting in 36 deaths and 49 injuries; In itaewon, South Korea, in 2022, 300 people were squeezed into an alley with a space of only 18 square meters, resulting in a total of 158 deaths. This incident has once again attracted international attention to the Highly Aggregated Tourist Crowds(HATCs). During the first May Day holiday since China optimized its COVID-19 pandemic response measures, certain tourist destinations witnessed an overwhelming surge in visitors. How to warn and prevent HATCs has also become an urgent problem to be considered and solved.

With regard to the study of crowd safety, extensive research has been conducted on crowd behavior, including analyses on the modes of pedestrian movement among different social groups, the behaviors exhibited by members of social groups during evacuations, and the behaviors of social groups under varying levels of crowd density [3]. The risk of overcrowding and potential trampling incidents is intimately associated with the escalation of crowd density, with extremely high density being a contributing factor to crowd-related disasters [4]. Thus, the examination of crowd density has also emerged as a salient aspect within the realm of tourism safety. Fruin noted that a density exceeding 7 individuals per square meter poses significant danger [5]. Nicholson concluded that the critical density of trampling accident is about 5 people per square meter through case analysis, but the critical density of squeezing accident is even higher, about 10 people per square meter, which may occur in almost static people [5]. In order to prevent the occurrence of tourism safety accidents, estimating and predicting the density of people in time is helpful to better manage activities and ensure public safety [6, 7]. Therefore, this study takes a specific group of tourists(HATCs) as the research object, which refers to more than 50 tourists in a local space with higher than 2.0 people /*m*^2^ crowd density [7–9]. And the focal point of this investigation centers around areas with HATCs, typically encompassing destinations for tourists to congregate, places hosting large-scale tourist events, ticket vendors situated in scenic areas, tourist information centers, terminals for cable cars (or sightseeing buses), tourist shopping quarters, hubs for transportation within scenic areas, and key connecting points (e.g., popular tourist spots) [9].

There are numerous techniques available for assessing crowd density, including counters, differential weight counters, infrared beams, wireless fidelity, and counters based on wireless sensor networks [10–14]. However, these methods suffer from limited accuracy and may prove inadequate for large-scale multi-directional or chaotic crowd movements [14]. According to the findings of Al-Zaydi *et al*. [15], the computer vision-based approach stands as one of the most viable options owing to the widespread adoption of cameras [16]. Over the past decade, the rapid advancements in crowd counting technology have demonstrated the potential of merging computer vision with artificial intelligence [14]. Efficient crowd control and management have become prominent areas of focus in the realm of intelligent video surveillance [17]. Nevertheless, there remains a dearth of comprehensive investigations into real-time monitoring and early warning systems for ensuring safety density among Highly Aggregated Tourist Crowds(HATCs).

In this study, a VGGT-Count network model was proposed to estimate the crowd density in four scenes of HATCs. Initially, the VGG-19 network received the crowd image as input for convolution. Subsequently, the transformer encoder with multi-head attention function received the flat output feature map. Ultimately, the density map was predicted using a regression decoder, which allowed for the differentiation of the level of crowding. Using an intelligent analysis model, real-time assessment of crowd density becomes feasible, enabling the determination of the crowd’s status based on predefined threshold parameters. Therefore, early warning can be conducted to help relevant departments implement crowd control and evacuation measures, such as capacity control, evacuation management measures, emergency evacuation routes, etc. In this study, the prediction model was used to innovate the detection method for HATCs in tourism, which is conducive to the risk prevention in advance in China’s tourist attractions and tourism management departments, reducing the occurrence of high-concentration safety accidents, and thus better promoting tourism development.

## 2 Related works

### 2.1 Safety of highly aggregated tourist crowds

Ensuring the safety of tourists in popular destinations is a pivotal aspect of their overall experience as well as a determining factor for the triumph of the particular locale [18]. Scholars are dedicated to studying the risk characteristics, influencing factors, and management strategies of Highly Aggregated Tourist Crowds (HATCs). Regarding risk characteristics, HATCs have temporal and spatial security risks. It has been observed that in China, the security risks of HATCs are particularly high during holidays, and their spatial distribution is expanding [19]. The main types of risks faced by HATCs include natural disasters, public health issues, accidents, social security concerns, crowd gathering risks, and space-related risks [20]. Overcrowding poses the greatest danger to crowds, including crowd surge, collapse, and trampling [21]. As crowd density increases, walking speed decreases, and the maximum possible flow (capacity) is reached at a moderate crowd density [22]. Thus, maintaining a certain range of density ensures the safety of tourists. In terms of influencing factors, Yin *et al*. [19] proposed that the safety of HATCs is affected by factors such as passenger flow pressure, tourists’ behavior status and strengthened management response. As a dynamic system, the number and density of tourists gathering in a specific space, exceeding its maximum capacity, create pressure on the tourist group, which becomes the basis for safety accidents. Insufficient or inadequate management response exacerbates the occurrence of safety accidents [20]. Alabdulkarim *et al*. [23] defined crowd management as the practice of controlling crowd activities before, during, and after events, including handling all elements such as personnel, venues, facilities, data, and technology. In terms of management strategies, scholars have studied various aspects such as sociology, psychology, and computer science, including crowd evacuation [24], crowd behavior [25–28], and crowd modeling [29–32]. Traditional crowd management strategies need to be integrated with technological means to provide accurate crowd-related information for optimal management [33].

### 2.2 Crowd counting

To mitigate and avert hazards, scholarly communities increasingly emphasized strategies for surveilling and assessing the perils associated with assembling masses [3]. Safety of crowd gathering is related to the number of people. Traditional crowd counting methods are divided into three categories: detection, regression, and point supervision. Detection techniques create its models [34, 35] to estimate the bounding box for each individual captured within the image. The anticipated density value count was given in terms of the number of bounding boxes. However, the occlusion of packed places and the requirement for extra annotations restrict its performance. Recent studies have focused on enhancing crowd count advancements through the utilization of regression-based techniques [36]. These methods involve creating pseudo-density maps using point annotations, resulting in accurate count forecasts. To further improve accuracy, advancements in multi-scale mechanical models [37] and perspective estimates [38] have been explored. To address the issue of erroneous pseudo-mapping creation, researchers have proposed alternative approaches. One such approach involves directly employing point hyper dimensionality, thus avoiding the potential errors associated with pseudo-mapping [38]. This alternative method has gained significant attention in recent years due to its potential to eliminate inaccuracies commonly associated with the creation of pseudo-density maps. BL [39] created loss functions by using Bayesian theory to compute each population’s predicted deviation.

### 2.3 Computer vision models

In recent years, Convolutional Neural Network (CNN) has been widely used in the field of crowd counting. CNN models usually count crowds by extracting features from images. These characteristics include information such as density, distribution and scale of the crowd. VGG network is a classic CNN model. Shen *et al*. [40] conducted research on estimating crowd image density using the VGG model. They further analyzed the number of individuals in the crowd and obtained positive outcomes. However, limitations arise when the VGG model is applied to long sequence data, prompting researchers to explore alternative models for crowd counting. Among these models, the Transformer has garnered attention due to its success in natural language processing and machine translation. For instance, Qian *et al*. [41] proposed a crowd counting approach based on the Transformer model in their investigation, enabling consideration of both global and local crowd characteristics. Additionally, the application of Visual Transformer (ViT) [42] demonstrates impressive performance through the implementation of transformer design specifically in visual contexts. Lin *et al*. [43] proposed a Multi-faceted Attention Network (MAN) to improve the Transformer model in local spatial relation coding. Tian *et al*. [44] used Pyramid Vision Transformer skeleton to capture the global crowd information, used Pyramid Feature Aggregation (PFA) model to combine low-level and high-level features, and used the efficient regression head of Multiscale Dilated Convolution (MDC) to predict the density map. Gao *et al*. [45] proposed an Dilated Convolutional Swin Transformer (DCST) for crowded scenes to achieve accurate positioning in high-density crowd scenes. Panboonyuen *et al*. [46] designed YOLOX and FPN decoders based on transformer architecture to effectively identify road assets in surveillance image sequence of Thai expressway.

In crowd counting, Transformer shows high performance, which is widely used in many practical scenarios. For instance, to uphold public safety and ensure efficiency of public spaces, it is crucial to apply video surveillance and traffic monitoring systems to constantly monitor and tally the movement of individuals in real-time. Moreover, during critical situations such as public events or natural calamities, the ability to accurately count crowds can aid emergency management agencies in swiftly responding and implementing essential protocols. Despite the broad application of transformers across various domains, the domain of tourism safety still lacks substantial exploration in the realm of crowd counting. Especially in the case of high crowds, such as scenic spots or concerts. It is of great practical significance to accurately count and warn in high-density and complex scenes. Hence, this study employed VGG architecture for feature extraction and constructed the VGGT-Count framework by integrating transformer encoder with a multi-attention mechanism. Aiming at the special group of HATCs, we investigated crowd counting and early warning method, which can not only extend insights on crowd counting in the field of tourism safety, but also provide more accurate and effective decision support for emergency management, thus providing more reliable technical support for tourism safety guarantee.

## 3 Method

### 3.1 Framework overview

**Fig 1:**
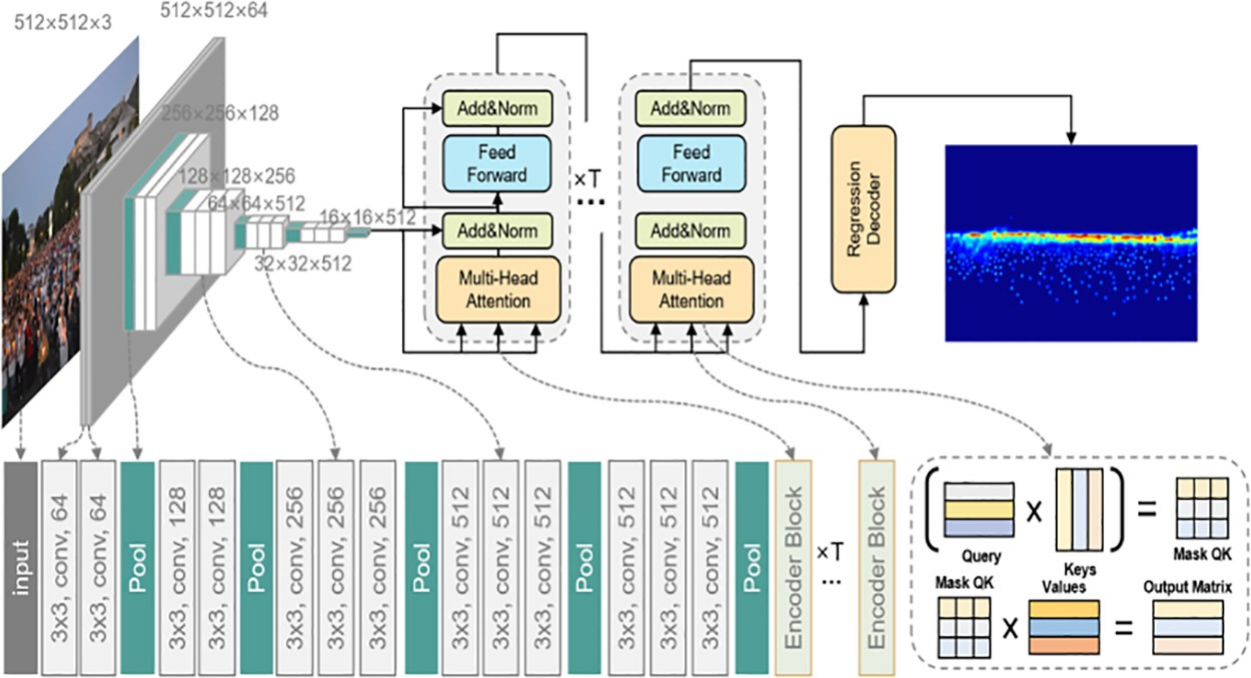
Analysis of loss variation in different epoch for a VGGT-Count network. A crowd image is first fed into VGG-19 network for convolution. Then the flatten output feature map is transmitted into the transformer encoder with Multi-Head Attention. Finally, a regression decoder predicts the density map. The Optimal Transport (OT) and Total Variation (TV) loss function is optimized during the training process.

The framework is illustrated in Fig 1. To begin, the features *FϵR*^*C*×*W*×*H*^ were extracted for each image *i* using the VGG-19 [40] as our backbone. These features were extracted based on the channel (*C*), width (*W*), and height (*H*) of the image. After flattening, the feature map was sent to transformer encoder, which incorporated multi-head attention to acquire features *F*′ across different scales. Following this, a regression decoder was employed to forecast the ultimate density map *DϵR*^*W*′×*H*′^ from the acquired features. Ultimately, we applied local attention regularization to effectively oversee the self-attention module training while also utilizing instance attention loss to constrain the total network training process.

### 3.2 Transformer encoder

The transformer encoder consists of a stack of *N* = 4 identical layers with each has two sub-layers. The first part is a multi-head self-attention mechanism, while the second is a simple feed-forward network with fully coupled and position-wise operation. After layer normalization, we used a residual connection around each of the two sub-layers. In other words, the function that the sub-layer itself implements was denoted as *Sublayer*(*x*), and the output of each sub-layer was *LayerNorm*(*x* + *Sublayer*(*x*)). All model sub-layers as well as the embedding layers generated outputs of size *d* = 512 in order to support these residual connections.

#### 3.2.1 Self-attention

The encoder in the Transformer network [42] utilized a self-attention layer. This layer facilitated connection between input and output positions, enabling consideration of global relations in current features. The computation of this layer involved connecting all pairs of input and output positions. It was computed by:

$$\begin{array}{c} Att\left(Q,K,V\right)=Sm\left(\frac{\left(QW^{Q}*{\left(KW^{K}\right)}^{T}\right)}{\sqrt{d_{k}}}\right)*VW^{V}. \end{array}$$ (1)

In this case, the scaling factor $\frac{1}{\sqrt{d_{k}}}$ was determined by vector dimension *d*, and *Sm* represented the softmax function. The weight matrices for projections were *W*^*Q*^, *W*^*K*^, and $W^{V}\epsilon R^{d*d}$. The query, key, and value vectors were represented by variables *Q*, *K*, and *V*, which were obtained from the source features.

#### 3.2.2 Multi-head attention

We discovered that it was more advantageous to linearly project the queries, keys, and values *h* times using various, learnt linear projections to *d*_*q*_, *d*_*k*_, and *d*_*v*_ dimensions, respectively, rather than executing a single attention function with *d* dimensional keys, values, and queries. After executing the attention function concurrently on each of these predicted iterations of the questions, keys, and valuations, we obtained *d*_*v*_ dimensional output values. The final values were obtained by concatenating and reprojecting them. The model may concurrently attend to data from several representation subspaces at various places due to multi-head attention. We used the attention weight *W*^*Q*^ to average the values, and further accessed to each position or output. By splicing that of all attention heads, we got the final multi-head attention output. The form was given by

$$\begin{array}{c} H_{i}=Att\left(QW_{i}^{Q},KW_{i}^{K},VW_{i}^{V}\right) \end{array}$$ (2)

$$\begin{array}{c} MultiHead\left(Q,K,V\right)=Concat\left(H_{1},H_{2},\ldots ,H_{i}\right)W^{O} \end{array}$$ (3)

where the projections were parameter matrices $W_{i}^{Q}\epsilon R^{d*d_{q}}$, $W_{i}^{K}\epsilon R^{d*d_{k}}$, $W_{i}^{V}\epsilon R^{d*d_{v}}$, $W^{O}\epsilon R^{hd_{v}*d}$. Each head computed associative relationships within different receptive windows in parallel. A block partitioning scheme was employed where separate blocks process the input feature map versions with different downsampling ratios, facilitating cross-scale interaction and fusion. Residual connections were adopted when merging the outputs of different blocks, allowing each scale to guide yet preserve the uniqueness of others and avoid information loss. Position encodings were embedded before restoring the input sequences into feature maps at multiple resolutions. This aided in reconstructing fine-grained details. During decoding, upsampling and downsampling modules were included to progressively rebuild high-resolution feature maps. By leveraging multi-head attention, block partitioning and the decoding solution, the proposed Transformer encoding architecture elegantly addressed the challenge of missing small object clues when dealing with multi-granular representations.

#### 3.2.3 Feed-forward

Every layer of our encoder and decoder had a fully connected feed-forward network, which was applied to each position independently with the same way besides attention sub-layers. This was comprised of a ReLU activation sandwiched between two linear transformations.

$$\begin{array}{c} FFN\left(x\right)=max\left(0,xW_{1}+b_{1}\right)W_{2}+b_{2}. \end{array}$$ (4)

In contrast, the linear transformations employed distinct parameters depending on the layer, even though they remained the same at different places. This can also be expressed as two convolutions with a kernel size of 1. The inner layer had dimensionality *d*_*ff*_ = 2048, while the input and output had dimensionality *d* = 512.

### 3.3 Loss function

We used three different loss functions in VGGT-Count: the counting loss, the Optimal Transport (OT) loss, and the Total Variation (TV) loss. The initial calculation entailed directly determining the disparity between the forecasted quantity and the actual quantity, while the subsequent two assessed the variation in the distribution of the normalized density function. Let *z*_*i*_ represent the vectorized binary map for dot-annotation, and let ${\hat{z}}_{i}$ represent the vectorized forecasted density map returned by a neural network. *z*_*i*_ and ${\hat{z}}_{i}$ were unnormalized density functions that can be used to produce three different loss functions.

#### 3.3.1 The counting loss

The objective of crowd counting was to minimize the disparity between *z*_*i*_ and ${\hat{z}}_{i}$, and the loss in counting was determined by the absolute difference between these two values:

$$\begin{array}{c} Loss_{c}\left(z_{i},{\hat{z}}_{i}\right)=\sum_{1}^{N}|z_{i}-{\hat{z}}_{i}|. \end{array}$$ (5)

where *N* was the number of training images.

#### 3.3.2 The optimal transport loss

We can transform both *z*_*i*_ and ${\hat{z}}_{i}$ unnormalized density functions into probability density functions by dividing them by their total mass. Accordingly, the OT loss was defined as follows:

$$\begin{array}{c} Loss_{OT}\left(z_{i},{\hat{z}}_{i}\right)=\lambda_{1}(\frac{z_{i}}{\sum_{1}^{N}z_{i}},\frac{{\hat{z}}_{i}}{\sum_{1}^{N}{\hat{z}}_{i}}). \end{array}$$ (6)

where the loss coefficient was λ_1_. The model benefits from the loss of OT since it can reduce the distribution gap between the ground truth and the anticipated density map.

#### 3.3.3 Total variation loss

Wang *et al*. (2020) [47] argued that the approximation of sparse crowd areas was not effectively achieved by OT loss alone. To address this issue, they proposed the incorporation of an additional TV loss for stabilization purposes. The TV loss can be represented as follows:

$$\begin{array}{c} Loss_{TV}\left(z_{i},{\hat{z}}_{i}\right)=\lambda_{2}\sum_{1}^{N}(\frac{z_{i}}{\sum_{1}^{N}z_{i}}-\frac{{\hat{z}}_{i}}{\sum_{1}^{N}{\hat{z}}_{i}}) \end{array}$$ (7)

where λ_1_ and λ_2_ were tunable hyper-parameters for the OT and TV losses. In order to guarantee that the loss from TV was proportional to the loss from counting, we multiplied the overall count by this loss term.

## 4 Experiment

### 4.1 Implement details

In this experiment, we used the pre-trained VGG-19 CNN backbone network, which was trained on ImageNet. For an analysis of the transformer encoder’s structure, we suggested referring to [42]. We replaced the attention module with our unique self-attention module to guarantee uniqueness. Because our self-attention module was built with spatial awareness, location encoding was not necessary when feeding the feature map directly into the encoder. An upsampling layer and three convolution layers with activation ReLU functions constituted our regression decoder. The final layer’s kernel size was 1 × 1, while the first two were 3 × 3.

For every training image, we first used random scaling and horizontal flipping. Next, we arbitrarily cropped picture patches in ShanghaiTech B and UCF-QNRF, each with a size of 512 × 512. Due to the presence of lower-resolution images in ShanghaiTech A, the dataset required a crop size adjustment to 256 × 256. Additionally, in all datasets, we restricted the shorter side of each image to a maximum of 2048 pixels. We adjusted the number of encoder layers (T) to 4 and the loss-balanced parameters (λ_1_ and λ_2_) to 100 and 0.1 respectively in order to maintain consistency. More appropriately, we used Adam [48] with a batch size of 1 to guarantee efficient training of transformer-based models. 1e-5 was the initial learning rate. 0.0001 L2 regularization was used to prevent over-fitting. PyTorch was used for all experiments, and a single 6G RTX2060 GPU was used.

### 4.2 Datasets

**ShanghaiTech Part A**. According to Zhang *et al*. (2016) [48], there are 182 images in the test set and 300 images in the training set. These pictures were taken at random from the web. And there is a huge variation in the quantity of people in these pictures. We got far better outcomes with additional color information and training data.

**ShanghaiTech Part B**. It contains 316 test photos and 400 training images that were collected by security cameras on Shanghai’s streets (Zhang et al., 2016) [48]. There are notable differences in crowd density and scale throughout these photographs. However, the converter-based backbone’s contextual modeling capabilities make it easy for VGGT-Count to capture these characteristics.

**UCF QNRF**. Dataset consists of 1,535 images with 1,251,642 header annotations overall (Idrees and Tayyab 2018) [49]. A training set of 1,201 photos and a test set of 334 images were created from these images respectively. Compared to the current population dataset, this dataset has more labeled heads, and a sizable part of the entities in the photos are small in size. Despite the abundance of small-scale objects present in this dataset, our model has the capability to effectively extract features from these diminutive entities.

### 4.3 Evaluation metrics

We evaluated various approaches for crowd counting by adhering to the convention of previous works [47]. The absolute error (MAE) and the mean squared error (MSE) were defined as follows:

$$\begin{array}{c} MAE=\frac{1}{M}\sum_{1}^{M}|z_{i}-{\hat{z}}_{i}|. \end{array}$$ (8)

$$\begin{array}{c} MSE=\sqrt{\frac{1}{M}\sum_{1}^{M}{\left(z_{i}+{\hat{z}}_{i}\right)}^{2}}. \end{array}$$ (9)

**Fig 2:**
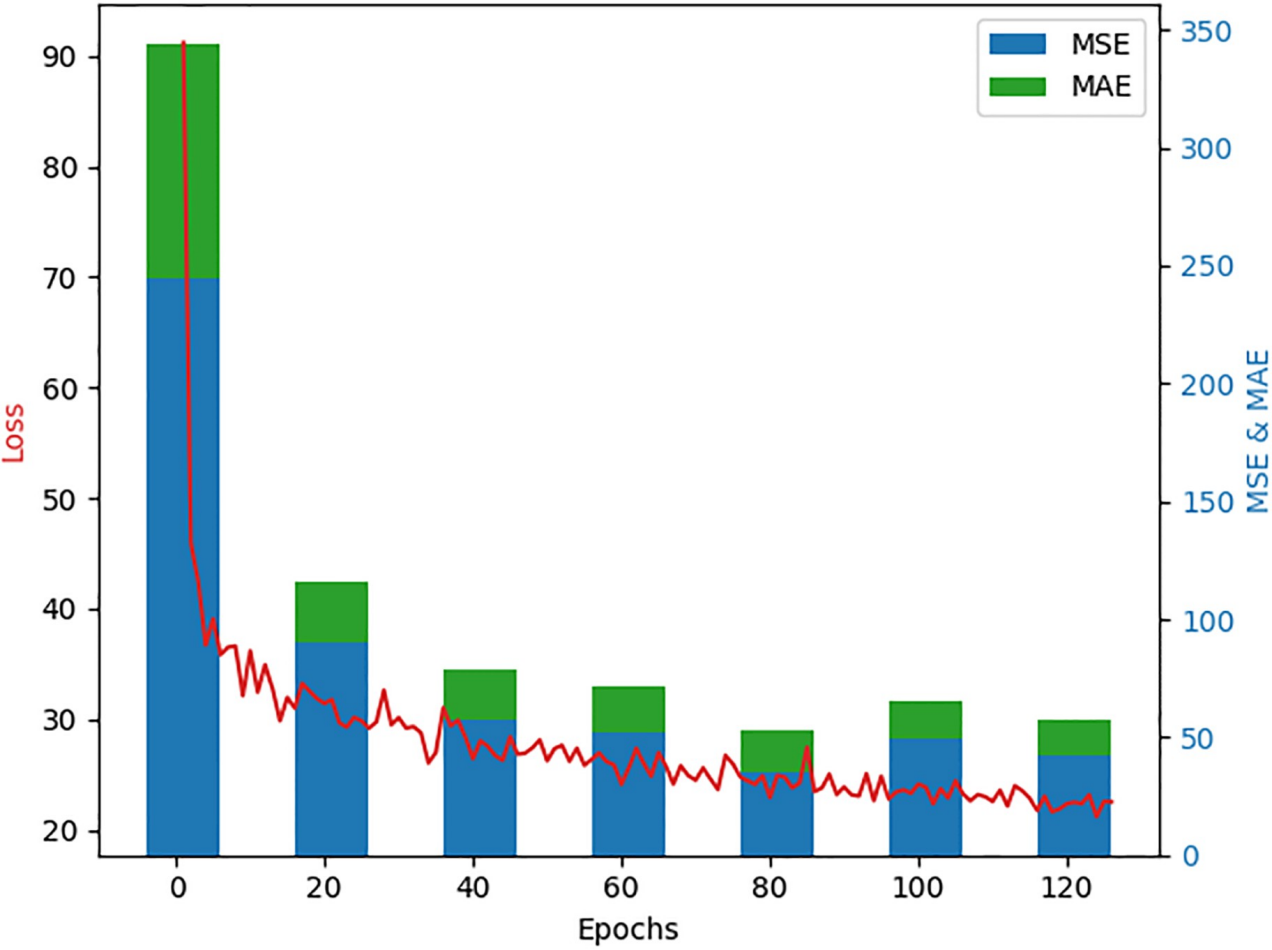
Analysis of loss variation in different epoch for a VGGT-Count network.

In the *i*th image, *z*_*i*_ indicated the exact number of people, whereas ${\hat{z}}_{i}$ denoted the estimated number of people, and *M* denoted the total number of test photos. MAE roughly represented the accuracy of the estimations, while MSE roughly showed the robustness of the estimates. In Fig 2, the density map across three datasets is displayed for visualization. Our research demonstrated the efficacy of VGGT-Count in effectively handling images from diverse sources and colorspaces.

The training results over 199 epochs are tracked in Fig 2. In the initial epochs, the loss saw significant reductions from 91.18 down to 36.72, indicating high learning progress as the model was adapting. As training continued, the loss gradually declined at a slower pace but maintained a downward trend, reaching 20.15 at the final epoch, demonstrating effective learning. The MSE and MAE metrics followed a consistent downward trajectory aligned with the loss. Notably, MAE exhibited the largest reductions, suggesting improvements to the model’s prediction precision for classification or regression tasks. Training time per epoch stayed stable around 131-132 seconds with no signs of expansion or contraction, reflecting steady computation resource usage throughout. Some tweaks could potentially yield better performance. Given the slowing loss rate evident in the figures, increasing the epoch count or decrementing the learning rate in later epochs may lead to finer-tuned results. Parameter tuning of batch size, optimizer, etc. also offers opportunities to enhance model quality. In summary, these figures provided a comprehensive view of key aspects in the training process, including loss and metric behaviors, computational efficiency, and potential optimization avenues—serving as a solid reference for reporting experimental findings.

### 4.4 Comparison with other methods

We evaluated our model on below three datasets and listed eleven recent state-of-the-arts methods for comparison. Our baseline is CCTrans [44], and we presented the quantitative results of counting accuracy in Table 1. As depicted in the results, our VGGT-Count achieved impressive accuracy across all three benchmark datasets. VGGT-Count surpassed the second-best method, CCTrans [44], by reducing the MAE value from 92.1 to 82.8 and the MSE value from 158.9 to 142.3. On the ShanghaiTech A dataset, our method achieved a MAE of 70.0 and MSE of 115.6, outperforming early methods like MCNN [48] and CSRNet [36]. While not achieving the best scores, it surpassed recent models like DM-Count [47] and DPN-IPSM [50]. The MAN model [43] obtained the best MAE of 56.8 and MSE of 90.3 on this dataset. On the ShanghaiTech B dataset, VGGT-Count achieved a MAE of 7.3 and MSE of 10.5, ranking second to P2PNet [51] based on these metrics. It outperformed other competitive models like SANet [37], DF-CNN [52] and UOT [53]. On the challenging UCF-QNRF dataset with variable crowd densities, VGGT-Count achieved a MAE of 82.8 and MSE of 142.3, ranking second to MAN [43] in performance. It surpassed methods such as MCNN [48], CSRNet [36] and DM-Count [47].

**Table 1:** Comparison with the state-of-the-art methods on ShanghaiTech A, ShanghaiTech B, and UCF-QNRF. The top performance is highlighted in bold, while the second best is underlined.

Method	ShanghaiTech A		ShanghaiTechB		UCF-QNRF	
	MAE	MSE	MAE	MSE	MAE	MSE
MCNN [48] (CVPR 16)	110.2	173.2	26.4	41.3	277.0	426.0
CSRNet [36] (CVPR 18)	68.2	115.0	10.6	16.0	-	-
SANet [37] (ECCV 18)	67.0	104.5	8.4	13.6	-	-
DF-CNN [52] (J COMPUT INT SYS 21)	-	-	-	-	218.2	357.4
DS-CNN,SS-CNN [54] (ARAB J SCI ENG 20)	-	-	-	-	115.2	175.7
DPN-IPSM [50] (ACMMM 20)	58.1	91.7	-	-	84.7	147.2
DM-Count [47] (NIPS 20)	59.7	95.7	7.4	11.8	85.6	148.3
UOT [53] (AAAI 21)	58.1	95.9	-	-	83.3	**142.3**
P2PNet [51] (ICCV 21)	**52.7**	**85.1**	**6.3**	**9.9**	85.3	154.5
CCTrans [44] (2021)	64.4	95.4	**7.0**	11.5	92.1	158.9
MAN [43] (CVPR 22)	**56.8**	**90.3**	-	-	**77.3**	**131.5**
**VGGT-Count(Ours)**	70.0	115.6	**7.3**	**10.5**	**82.8**	**142.3**

As shown in Table 2, DM-Count, which has the largest model size of 72.5M, achieved the slowest prediction speed. Its frame rate is only 52.4 fps with inference time up to 202 milliseconds. This is mainly because DM-Count adopted a deep CNN network, requiring a large number of parameters and computational resources. Compared with DM-Count, DS-CNN and SS-CNN reduced their model sizes to 57.0M but only achieved a frame rate of 54.4 fps and inference time of 180 milliseconds, without significant improvement. This could be attributed to their still deep network structures. In contrast, MAN and CCTrans introduced Transformer structures into their designs, making the models lighter at 30.9M and 29.9M, respectively. Consequently, their prediction speeds were substantially enhanced, with frame rates reaching 58.2 fps and 58.0 fps. However, due to structural differences, CCTrans achieved a lower inference time of 108 milliseconds. Our proposed VGGT-Count model employed a lighter VGG network as the backbone combined with Transformer modules, achieving an even smaller model size of 30.2M. Importantly, it maintained a high frame rate of 58.8 fps while reducing the inference time to 120 milliseconds, closing the gap to MAN and CCTrans. This demonstrates that our hybrid design optimized both predictive performance and computational efficiency given a relatively compact model capacity.

**Table 2:** Comparison of real-time performance in different models with size, frames and inference time.

	Model Size (M)	Frames/s(fps)	Inference time(ms)
DM-Count [47]	72.5	52.4	202.0
DS-CNN, SS-CNN [54]	57.0	54.4	180.0
CCTrans [44]	29.9	58.0	108.0
MAN [43]	30.9	58.2	113.0
**VGGT-Count(Ours)**	30.2	58.8	120.0

### 4.5 Ablation studies

This study systematically investigated the role of different model components in sequence prediction tasks as shown in Table 3. Firstly, when VGG19 was used solely for feature extraction and classification, the model achieved MAE and MSE of 12.1 and 14.0 respectively, indicating low prediction accuracy due to CNN’s limited capability in capturing long-range dependencies. The Transformer encoder-decoder structure was then established as the baseline model, being observed a significantly improved performance with MAE and MSE decreasing to 10.6 and 12.5 respectively. This verifies Transformer’s powerful sequence modeling ability through leveraging multi-head self-attention to capture global dependencies across all input positions. Subsequently, experiments with varied numbers of attention heads were conducted to assess their impacts. As the number of heads increased from 4 to 8, the performance continually increased as evidenced by the declining MAE and MSE curves. This suggests that multi-head attention can learn representations from different subspaces, thereby enhancing the model’s representation capacity. Lastly, introducing the Counting Loss and Total Variation Loss functions achieved further improvements through fine-tuning. Particularly, Total Variation Loss led to the largest performance gain with over 0.5 drop in both MAE and MSE, demonstrating its effectiveness in alleviating blurry predictions. In summary, this set of rigorous ablation tests unveiled the optimal design of combining VGG features with Transformer encoder-decoder framework for sequential prediction tasks. The findings provide valuable guidelines for modeling similar problems hereafter.

**Table 3:** Optimizing performance by using different components and structures on ShanghaiTech B datasets.

Component	Combinations				
VGG19	✓			✓	✓
Transformer		✓	✓	✓	✓
Multi-Head Attention(N = 4)		✓			
Multi-Head Attention(N = 8)			✓	✓	✓
Counting Loss	✓	✓	✓	✓	
Total Variation Loss					✓
MAE	12.1	10.6	9.3	7.8	**7.3**
MSE	14.0	12.5	12.0	11.2	**10.5**

## 5 Discussion

### 5.1 More results analysis

**Fig 3:**
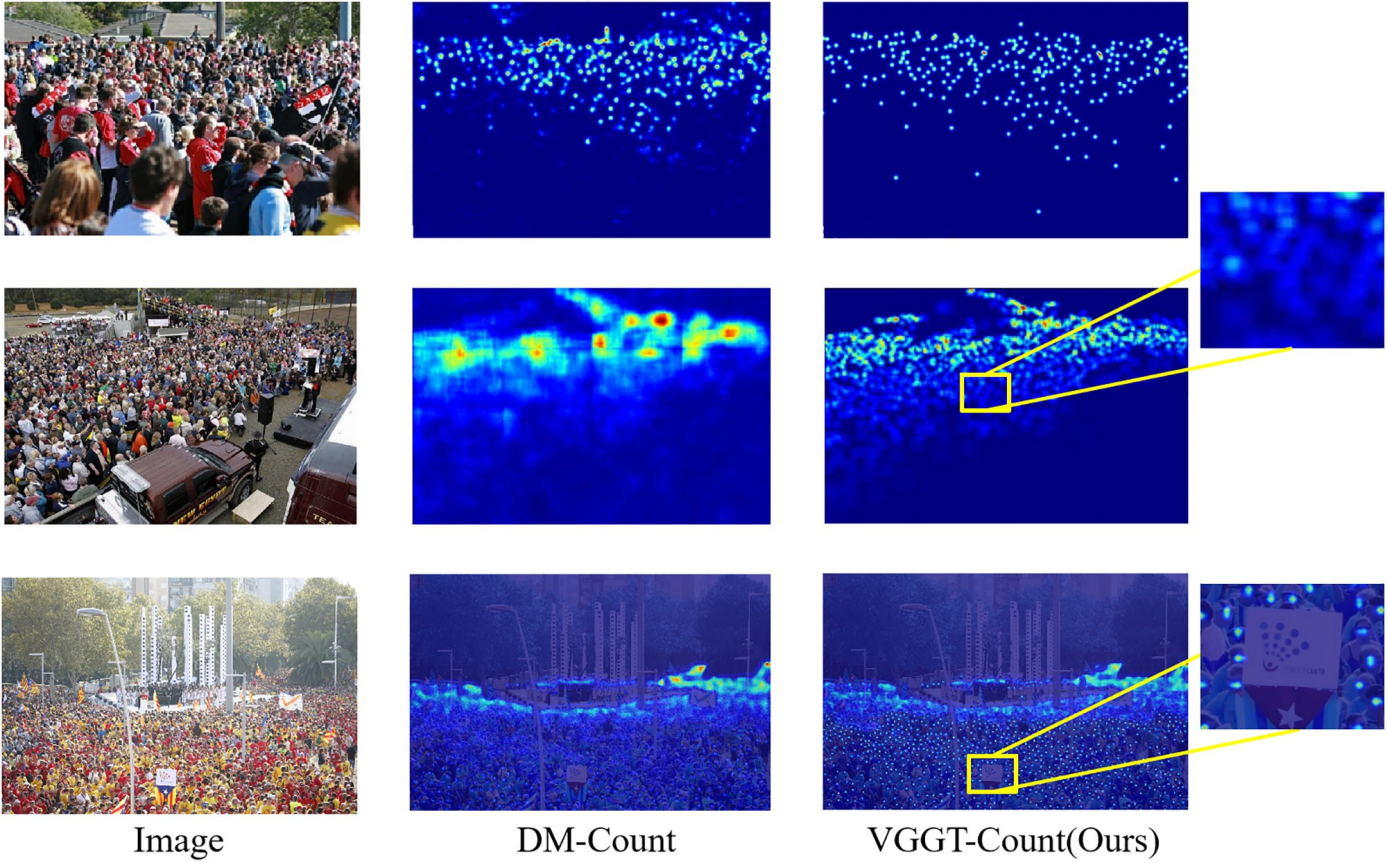
Visualization results of VGGT-Count vs DM-Count.

We provide additional comparison on experimental results in this section. As shown in Fig 3, compared with DM-COUNT model, the key advantage of VGGT-Count model regarding to crowd counting prediction results lies in its utilization of Transformer attention mechanisms to better capture the relationships and differences between different image regions. Specifically, when the crowd distribution is uneven (some areas are densely populated while others are sparse), DM-COUNT is prone to ignoring differences between regions, leading to overcounted or undercounted values. VGGT-Count leverages attention to reflect population density variations more accurately across regions. At the same time, when some areas are obstructed, it is of difficulty for DM-COUNT to generate accurate counts, while VGGT-Count can learn contextual information surrounding the obstructed region to enhance prediction performance. VGGT-Count is able to extract fine-grained regional details to provide more stable and flexible data fitting results with adaptable tolerance. Overall, through exploiting multi-head attention mechanisms, VGGT-Count has an advantage in identifying distinct regional features and capturing inter-regional relationships. This enables it to generate more reliable and precise counting predictions under complex real-world conditions involving uneven crowd distributions and density fluctuations.

In addition, two unsuccessful results can also be obtained from Fig 3. On the one hand, with limited ability to process low-resolution images, it may be difficult for VGGT-Count model to accurately identify and count individuals in low-resolution images because the characteristics and details of individuals become less obvious and more indistinguishable. This may result in inaccurate counting, and thus reduce the overall performance in a low-resolution image environment. On the other hand, occlusion prediction is full of challenges as model may face difficulties predicting the number of individuals obscured or hidden by other individuals or objects in the image. This is due to its dependency on features and spatial information within the image, yet the occluded individuals may be unable to provide sufficient information for the model to accurately predict its number. In a word, all of these can be the motivation for our subsequent optimization of the model and research.

### 5.2 Application and feasibility

To further explore the practical application and feasibility of the VGGT-Count network model, we would like to provide specific examples on how this model can be applied in real-world tourism management scenarios. Firstly, the VGGT-Count model can be used by tourism authorities to monitor and manage crowd density in popular tourist destinations. By deploying surveillance cameras and utilizing the VGGT-Count model, real-time crowd density information can be obtained. The collected data can be analyzed to predict the crowd distribution in major areas during peak hours, especially during holidays, popular scenic spots and major events, so as to issue early warning signals in time. The information can also assist in making informed decisions regarding crowd control measures, such as adjusting entry and exit points or implementing crowd diversion strategies. Secondly, the VGGT-Count model can be integrated into mobile applications or tourist information systems. This would allow tourists to access crowd density information for different attractions or areas in real-time. By providing this information, tourists can make informed decisions about their itinerary and choose less crowded locations, to receive a more enjoyable experience. In summary, the VGGT-Count network model has significant potential in the field of tourism management. By applying it to real-world scenarios, such as crowd monitoring in tourist destinations, the model can provide valuable insights and supports on crowd control measures.

## 6 Conclusion

**Fig 4:**
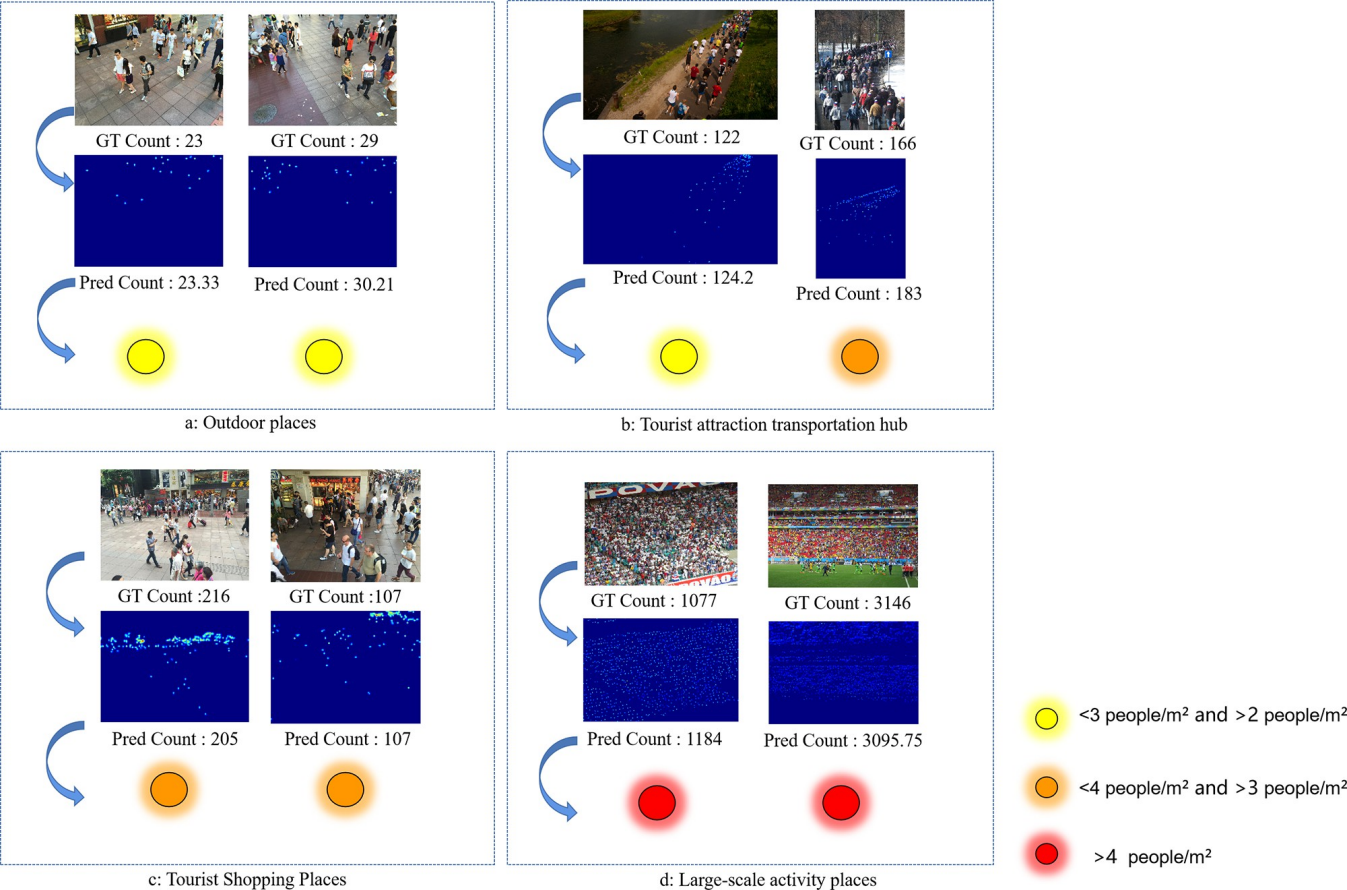
Visualization results of VGGT-Count in different scenarios.

In this study, we developed a network model, VGGT-Count, to estimate the number of people in high-density areas. This model enables real-time surveillance and forecasting of crowd density in HATCs locations incorporating an early warning system tailored to distinct density thresholds. And our method also performed best on the four tourist crowd scenarios(see Fig 4) The conclusion is as follows:

1. The experimental results show that the VGGT-Count network model proposed in this study has high accuracy on all three benchmark data sets.
2. The VGGT-Count network model is used to predict the density on four scenes of HATC sites. Early warning is realized according to the range of three different density thresholds. The accuracy and practicability of early warning can be enhanced by using this technique to subdivide scenes, which allows for a more precise reflection of the flow of individuals in various regions. Consequently, it enables meticulous counting and alerting of people, with improved accuracy and practicability of the warning system.

### 6.1 Contribution

Previous studies have focused on crowd counting in general scenarios. However, our research specifically addressed the challenges of estimating crowd density in high-density areas. By developing the VGGT-Count model, we extended insights on crowd quantification in such complex and crowded environments. Secondly, our proposed model incorporated an early warning system tailored to distinct density thresholds. This addressed the need for proactive crowd management and safety measures in HATCs. By accurately predicting crowd density and issuing timely warnings, our model can enhance the preparedness and response capabilities of authorities, facilitating effective crowd control strategies. Lastly, our research demonstrated the practical application and feasibility of the VGGT-Count model in real-world scenarios. By providing accurate crowd density estimates, our model can assist tourism management authorities in making informed decisions regarding crowd control measures and enhancing the overall tourist experience. In summary, the proposed VGGT-Count model, along with its early warning system, offered practical solutions to crowd management and safety in HATCs, thereby contributing to the advancement of the field.

### 6.2 Future work

Although this study has made some achievements in counting HATCs, it still has some limitations. Firstly, the generalization ability of this study needs further verification due to the relatively simple experimental data set, which fails to encompass a broader range of tourist sites. Secondly, in order to achieve the optimum counting effect, the VGGT-Count network model utilized in this research may require adjustments of various parameters for different high-concentration locations. The future development direction includes but is not limited to the following aspects: Firstly, other advanced crowd counting methods, such as deep learning and artificial intelligence, can be further explored to improve the counting accuracy and real-time performance. Secondly, we can consider using a variety of data sources for comprehensive analysis, such as sensors, webcams and other equipment, to collect data, and to improve the accuracy and stability of monitoring and prediction.
